# Physicians’ challenges when working in the prehospital environment - a qualitative study using grounded theory

**DOI:** 10.1186/s12245-024-00599-0

**Published:** 2024-02-27

**Authors:** Denise Bäckström, Aida Alvinius

**Affiliations:** 1 Department of Biomedical and Clinical Sciences, Linköping University, Linköping, 581 83 Sweden; 2 Department of Leadership and Command & Control, Swedish Defence University, Våxnäsgatan 10, Karlstad, 651 80 Sweden

**Keywords:** Prehospital, Physician, Doctor, Organization, Environment, Leadership, Emotional management, Communication, Grounded theory

## Abstract

**Background:**

In the rapid development in prehospital medicine the awareness of the many challenges in prehospital care is important as it highlights which areas need improvement and where special attention during education and training should be focused. The purpose of this study is to identify challenges that physicians face when working in the prehospital environment. The research question is thus; what challenges do physicians face when working in prehospital care?

**Method:**

This is a qualitative study with an inductive approach and is based on individual interviews. The interviews were analyzed using the Classic Grounded Theory (GT) method as an approach. The interviews were conducted as semi-structured interviews via the digital platform Zoom during winter / early spring 2022.

**Results:**

Challenges for prehospital physicians can be understood as a process that involves a balancing act between different factors linked to the extreme environment in which they operate. This environment creates unique challenges not usually encountered in routine hospital practice, which results in trade-offs that they would not otherwise be faced with. Their individual situation needs to be balanced against organizational conditions, which means, among other things, that their medical decisions must be made based on limited information as a result of the constraints that exist in the prehospital environment. They must, both as individuals and as part of a team, manoeuvre in time and space for decision-making and practical tasks. This theory of balancing different entities is based on four themes; thus the theory is the relation between the four themes: *leadership*, *environment*, *emotion management* and *organization.*

**Conclusions:**

With the help of previous studies and what we have found, it is reasonable to review what training is needed before starting to work prehospital as a physician. This should include components of the themes we have described: organization, environment, leadership and emotional management.

**Supplementary Information:**

The online version contains supplementary material available at 10.1186/s12245-024-00599-0.

## Background

Prehospital medicine has developed rapidly in recent decades from being a pure transport organization. The firefighters who then manned the ambulances received only a few weeks of medical training. The development continued and in the 90’s Sweden started to use nurses in ambulances, at about the same time the ambulance service transferred from being part of the rescue service to being part of the county councils (which today are the regions). The role of Ambulance nurse became a post graduate education pathway for registered nurses [1]. This development meant that not only were patients transported to the hospitals, but assessment and treatment began as soon as the ambulances reached the patient [1]. A continuation of this medical development is what we see today with specialist physicians working in support units that augment the ambulance service. It is usually an anesthesia and intensive care physician, which is the same type of physician who responds to alarms in hospitals when a patient is critically unwell.

The challenges in the prehospital environment differ from those found in hospitals. Ambulance personnel often experience stress. The dispatch process, insufficient resources and lack of information contribute to this stress whilst peer support and conversations reduce stress [2]. The approach to personal safety in prehospital staff may be poor, particularly when dealing with multiple casualties. This can even lead to staff not using safety equipment, even though it is available [3].

One of the key priorities for delivery of healthcare is to maintain patient safety, which means reducing the number of incidents and adverse events for patients. The interest in, and knowledge of, how non-technical skills (NTS) contribute to patient safety has increased in recent years as several studies with different methodologies have been able to show that poor teamwork strongly contributes to adverse events [4–6]. What actually is included in the term NTS is not entirely clear; it varies between different studies. There is also a difference between what is included within the medical literature in the field of NTS and what is highlighted in the field of social sciences. It has been found that published findings in one area do not reach the other area but do then subsequently also appear there [7]. One overall description of what NTS include is that they are the cognitive and interpersonal skills that complement an individual’s clinical skills in order to deliver good healthcare [8]. The vague definition of non-technical skills makes it difficult to define and assess these skills in clinical practice and results in a lack of standardization, difficulty to measure effectiveness and inadequate attention to non-technical skills.

In the rapid development in prehospital medicine the awareness of the many challenges in prehospital care is important as it highlights which areas need improvement and where special attention during education and training should be focused. The purpose of this study is to identify challenges that physicians face when working in the prehospital environment. The research question is thus; what challenges do physicians face when working in prehospital care?

## Method

This is a qualitative study with an inductive approach and is based on individual interviews. The interviews were analyzed using the Classic Grounded Theory (GT) method as an approach [9, 10]. The interviews were conducted as semi-structured interviews via the digital platform Zoom during winter / early spring 2022.

### Study population

The ten participants included in the study were selected with a convenience sample [9]. The research team opted for convenience sampling to efficiently gather data from participants who were readily available and easily reachable within the given time frame. This sampling approach was deemed appropriate for the scope of the study, allowing for a quick and cost-effective collection of data. The physicians who were included were found via contacts at the various prehospital bases in Sweden. They were contacted via email and selected based on availability during the interview period. There were no refusals or dropouts when selected based on availability. The interviews took 45 min to 1 h and 10 min, and saturation was established after eight interviews.

Physicians who work in the prehospital environment in Sweden are consultants, with those who were included in the study being anesthesia and intensive care physicians. No physician works completely in the prehospital environment, it is always only part of their work. This is because it is difficult to maintain clinical skills when working in prehospital care. The participants in the study work 20–50% prehospital, usually on a weekly basis. Half of the physicians in the study work in an ambulance helicopter service and half work in a rapid response car. The physicians working in rapid response cars work in more densely populated areas and the physicians working in ambulance helicopters work in more rural areas. The participants are between 43 and 63 years old and have worked between 1 and 31 years in prehospital care. One of the participants is a woman, which roughly corresponds to the distribution that can be seen among physicians working in prehospital care in Sweden.

### Study design

The individual interviews were conducted during the period 4 January to 18 March 2022 and were of a semi-structured nature with the help of an interview guide (Appendix 1). The interview questions were open and inductive to allow the informant to identify and formulate challenges themselves [9, 10]. The interview guide was developed with the aim of including all aspects of prehospital work and starting with a broader perspective and ending with more personal experiences. No pilot interview with the questions was conducted. When a respondent accepted participation in the study, he or she was given written participant information stating the purpose of the study and methodology. No questions or additional information beyond that provided in the participant information was disclosed to the participants prior to the interviews. Participants signed documents of consent to participate before interviews began. The interviews were conducted digitally using the video conferencing tool Zoom where both audio and video were used. Video was chosen to facilitate the interaction between the physician and the researcher. The video conference method was chosen to enable participants form different places in the Sweden to participate without making travels an obstacle. Participants did not provide feedback on the findings.

### Analysis

All interviews were transcribed before analysis began. In the open phase [9, 10], the quotations were coded before the interviews were printed in paper form. Codes were identified from quotes in the interviews and could consist of statements or descriptions of emotions. The printed transcripts with associated code were cut apart and sorted into different categories and after that themes (Table 1). The open phase ended when the material was considered saturated, and no further codes or categories could be identified. In the last theoretical phase [9, 10], connections between categories were sought in order to create themes and formulate a grounded theory of how themes and categories relate to each other. Field notes were taken during interviews and throughout the analysis process, which were considered in shaping the grounded theory.

Grounded theory was chosen to enable a detailed understanding and generation of new theories of the specific social and organizational concepts in prehospital work. GT emphasizes the emergence of themes and patterns from data, whereas for example reflexive thematic analysis often seek to identify pre-existing structures of frameworks.

**Table 1:** Categories and themes

Category	Theme
Leadership styles	Leadership
Reflexive leadership	
The team and task	
Decision and responsibility	
External environmental factors	Environment
Personnel and relational aspects	
Strategies for adapting to the environment	
Ethical and emotional stress	Emotional management
Emotional management	
Stress reactions	
Excitement attracts	
Organizational and structural aspects	Organization
Working conditions	
Knowledge and skills	

All the categories and how they were organized under different themes

### Reflexivity

To explain to the reader how the theory is connected from quotation to theory, we have described which different themes we have found, and which categories are included and illustrated them with selected quotations. In this study, the interviews were conducted by the less experienced researcher, who is a prehospital physician. The interviewer had a preunderstanding due to her own experience working in prehospital care but had no other motive behind the study than to gain more knowledge and understanding of the challenges faced by physicians in prehospital work. In order that the pre-understanding would not affect the respondents, open-ended questions have been asked. The respondents themselves have had to identify what could be considered challenges and it was interesting to find that the challenges almost exclusively related to non-technical skills. All steps from coding to formulation of theory have been carried out by two people, one of whom is an experienced researcher (associate professor in leadership and command and control) with knowledge of the method grounded theory and the other a beginner in this context (PhD in trauma), both are female. One has no previous experience from the environment or profession studied and the other is a prehospital physician. Both researchers have carried out the analysis together and this quality criterion is called the inter-assessor perspective [11]. This can be seen as a strength in that it reduces the risk that the pre-understanding affects the analysis to an excessive extent. Consensus was reached on all aspects from coding to formulating a theory, disagreement was solved after discussions.

### Ethical considerations

The participants were provided written information upon agreeing to participate in the study. Prior to the interview initiation, participants were verbally briefed, followed by the signing of written consent forms. Participants remained anonymous and had the option to withdraw their participation until the article’s submission. The recorded material is stored separately and is accessible only to the research group in compliance with the regulations governing research at Linköping University. The interviewer maintained transparency regarding their preconceptions both during interviews and in the drafting of the article.

The study has ethical approval from the Swedish Ethical Review Authority, number 2021-05550-01.

## Results

The results are presented with quotes from the participants, where participants are numbered from 1 to 10.

Challenges for physicians in the prehospital environment can be understood as a process that involves a balance between;

- organizational and individual factors linked to extreme environments.
- manoeuvre in time and space, both individually and in teams.
- the interaction between active and passive leadership, in extreme environments.
- one’s own and others’ emotional states.

This theory of balancing different entities is based on four themes; thus the theory is the relation between the four themes: *leadership*, *environment*, *emotion management* and *organization.* Challenges for prehospital physicians can be understood as a process that involves a balancing act between different factors linked to the extreme environment in which they operate. This environment creates unique challenges not usually encountered in routine hospital practice, which results in trade-offs that they would not otherwise be faced with. Their individual situation needs to be prioritized against organizational conditions, which means, among other things, that their medical decisions must be made based on limited information as a result of the constraints that exist in the prehospital environment. They must, both as individuals and as part of a team, manoeuvre in time and space for decision-making and practical tasks.

These conflicting challenges result in fluctuations between active and passive leadership with doctors needing to alternate between the two different methods in the extreme environment in which they operate. Leadership varies depending on the situation. Prehospital physicians are often surrounded by colleagues who are used to making decisions and so physicians can rapidly go from a passive to an active leadership style and then back to passive mode again during the same assignment. There seems to be a balance where they only take an active role when clearly required and as soon as there is an opportunity, they revert to a passive role again.

There is also a balancing act when it comes to their own and others’ emotional states. They take great responsibility for the feelings of the patient, relatives and the rest of the team both during the assignment but also after the assignment.

The four themes: *leadership*, *environment*, *emotion management* and *organization* that make up the components of the theory are further explained below.

### Theme: leadership

Leadership in a prehospital environment refers to the physician’s management of the work during an assignment. Looking at leadership, parts of followership also emerge and the challenges associated with making decisions. The theme of leadership contains four categories; *leadership styles, reflexive leadership, the team and task, decision and responsibility*.

The analysis of the interviews shows how the physicians work with different kinds of leadership styles depending on the situation and conditions. Several respondents describe that they avoid taking an active leadership role as long as the situation develops in a direction they wish. In the normal situation, physicians have an inclusive approach, but this changes when the situation becomes time-critical and then they move to an authoritarian leadership style. This seems to happen consciously and with the understanding that this can have negative consequences for teamwork. Below are two examples;

“Practically speaking, I usually initially, unless the situation requires a very urgent action, stay in the background…” (3).

“It’s usually about the patient being stabilized medically so that I do not have to be completely focused and active in the care anymore, so I like to hand over and it’s a lot about freeing up mental space for oneself, maybe taking a step back, think, have we done everything, is there anything more that needs to be done?” (5).

The analysis shows how the category *reflexive leadership* describes how the physicians reflect on their leadership in relation to the event they were involved in. All respondents describe how after a challenging event they have a conversation with the team about what happened and the majority of them also describe how after this conversation with the team, they have an ongoing process where they reflect on their decisions. They have an internal dialogue where they go through the event and think about the decisions that were made and how the situation developed. Some also use colleagues who did not participate in the event to continue to reflect on the situation they have been through.

“I try to be self-critical in a balanced way, because what didn´t go well you have to acknowledge it, it did not go quite the way I wanted. It did not go badly but it did not go quite the way I wanted and then you have to be able to say that, yes, I can do better.” (3).

The category *team* shows how the physicians relate to the temporarily composed teams that they work with in the prehospital environment. Despite the non-existent preparation time, it is clear that the physicians are trying to get to know the team and what abilities they have. There is a respect and balance of power within the temporarily composed team and the physicians try to support the other individuals in the team, among other things through how they communicate.

“I think I may be more inclined to ask questions than to give directives…” (7).

“Because prehospital activities are not just about medicine but about the ability to work in teams, it is about the ability to, in a bad situation with poor opportunities, try to communicate in a sensible way so that the team as a whole performs as well as possible… ” (1).

The category *task, decision and responsibility* sheds light on how physicians feel responsible for the overall situation at the same time as facing demands and expectations from the temporary team. There are also challenges when it comes to decisions, analysis and priorities. Decisions that need to be made on limited information when time is short are challenging. Limited information also contributes to the fact that it is a challenge to obtain an overview of the situation. Priorities permeate many of these decisions, including which patient should receive more resources and what needs to be done first for any individual patient.

“Mentally, I probably do not let go of the perception that I am leading the situation, but I can accept letting go of the detailed control many times …” (2).

“The prehospital situation is about very limited decision basis, you have to dare to make decisions …” (5).

### Theme: environment

The theme *environment* describes how the work is affected by the prehospital environment and what challenges it entails. There are also aspects of how other challenges affect the environment as leadership affects their psychosocial environment. The theme contains three categories; *external environmental factors, personnel and relational aspects* as well as *strategies for adapting to the environment.*

The category of *external environmental factors* contains sections about how the physical environment affects the work, for example weather and wind, but also factors such as threats and violence in the prehospital environment. In addition, there are challenges in the form of complexity where the respondents describe how they simultaneously need to evaluate different information at different levels, for example taking care of one seriously ill patient while they receive a report about another patient and have to prioritize where they are needed most. Rare incidents also involve a challenge that most of the respondent’s mention; a demand upon on knowledge and skills that are rarely used. Injured children are specifically mentioned here.

“It is often a difficult context to get a grip on as quickly as you need it and you do not have time to be very thorough…” (2).

“There are a lot of external factors, partly the environment, there can be different threats it can be difficult to get to the patient, it can be difficult to take the patient from there…” (10).

The category of *personal and relational aspects* addresses the psychosocial environment and culture of prehospital care where, among other things, one is not expected to express feelings after difficult events but also there is a strong community in which people put in extra effort to solve situations. Collaboration with other prehospital actors is also addressed here and the challenges in understanding each other. What dominates this category are the different aspects of working alone, how as a physician you can be left without realistic opportunities to get help from someone else with more knowledge or skills.

“So it was extremely loyal, mandatory stand up for each other, do what you did it was a bit of a culture of honor, that honor stretched to keep the car clean and behave outside of work.” (9).

“I have behaved a bit like the old tribe who brushed off the feelings and moved on and I usually joke, am I a psychopath or am I just very suitable for my profession …” (6).

“You have to have a huge margin on it because no one else will come if you need help, no matter what experience or skills you have, you must have a safety margin in your way of working.” (4).

The category of *strategies for adapting to the environment* includes consequences of the environment. There are limitations in prehospital care to what is medically achievable and environmental factors distract and complicate this work. This category also contains sections on how to optimize the conditions and highlights that there is habituation to all aspects of the prehospital environment.

“So it means more steps and less prepared steps with fewer people, I think that is the biggest challenge.” (7).

The environment affects the conditions for the physicians with the physical environment being challenging in many ways, including weather, location of events and physical threats. The psychological challenges of this environment are also something that attracts the physicians to prehospital care.

### Theme: emotion management

The theme of *emotion management* addresses how physicians need to navigate between various emotions, not only their own but also the emotions of the patient, relatives and the rest of the team. This theme contains four categories; *ethical and emotional stress, emotional management, stress reactions* and *excitement attracts*.

The category of *ethical and emotional stress* is described by all respondents as a challenge in the prehospital environment. Stress is highlighted both as a reaction to the patient’s and relatives’ feelings but also due to feelings about the patient’s life story. It describes how the hospital environment protects against much of this stress and that the white clothes and sterile environment mean that there is a distance from the patient in the hospital that is not there prehospital. The different stress levels of the members of the team also create challenges for the physicians who will try to lead the team. Finally, the solo physician role, with the responsibility that the physician carries results in stress.

Interviewees also describe how the challenges are something that attracted them to take a prehospital position and the majority also describe a “blue light romance” with an attraction to being able to drive a car quickly and be able to represent high competence prehospital care.

“the environment in hospitals is a bit protective, you do not have that protection outside, there it becomes rawer and more naked and the risk is quite high that you are not injured emotionally but that you are hurt in some way” (1).

“It’s like the protection you have, to be competent, to be able to handle things, it’s a little bit scary to be so alone.” (8).

The category *emotional management* mainly consists of two different areas, emotional management of oneself and emotional management of others, which can be divided into; the team, the patient and relatives. The respondents describe how they take great responsibility for the stress of the environment and how they try to handle both that and their own stress.

“One way to make the situation less challenging is to try to lower everyone’s stress level and your own or at least not show that you are stressed” (3).

“On the way to a scene, you kind of have to prep a little, yes, tell yourself that it will go well.“(7).

The category of *stress reaction* consists of the acute reactions that occur in association with the work and delayed reactions that affect the physicians for a long time after the event. There are both physical reactions such as palpitations and mental reactions such as inability to sort impressions or inability to express oneself in a constructive way.

“The heart palpitations will return, but it’s okay” (2).

“During 3–4 weeks that I actually slept badly from this case, woke up a few times at night, maybe once a night on average, sometimes not, sometimes twice and thought about it but after that it was all good and then it came back almost a year later ” (7).

The category of *excitement attracts* describes how the combination of riding a helicopter or riding a car quickly attracted people to work prehospital, but how this freedom that exists when it comes to decision-making becomes a challenge that creates stress. The balance between being a hero or a failure is subtle and the feelings around the different positions are the opposite.

“It is a freedom that comes with a responsibility in that environment that poses other challenges for yourself.” (6).

“You drove fast and so on, that was probably what got me interest initially, I can say, I must admit.” (5).

The emotions of the team, the patient, relatives of the patient and of course the emotions of the physicians need to be balanced so that the team can perform well. The emotional management is closely related to leadership, where the physicians lead the patient and the team by managing feelings.

### Theme: organization

In the theme *organization*, the analysis shows that organization entails challenges in the form of; *organizational and structural aspects, working conditions* and *knowledge and skills*.

The category *organizational and structural aspects* describe how decisions are made about when and how patients are to be transferred. These decisions are important in the bigger picture because they also affect other resources and other patients who need healthcare. The respondents also describe how the equipment used is often developed for use in hospitals, which means limitations when it is used in the prehospital environment. The respondents describe how it is not possible to bring all your equipment to the patient when working prehospital, which means that they often have to choose what they bring, which can cause constraints when they take care of patients. The equipment needs to be small and able to be used flexibly, and hence might have only the most basic functions, which in turn means that it might not fulfill its intended function and so risks not being used. Limited resources mean that the amount of equipment and the limited number of people working prehospital causes challenges. The small number of people available to take care of the patient means that there might be no one who can prepare for the next step and so everything takes longer.

“There is no one else who just does things so that you know that the next step is prepared.” (3).

“What the problem is, I think, is how much should you be able to carry while taking care of the patient? What is most important? Too often, I think you have to choose between monitoring and patient care.” (9).

The category *working conditions* shows that there is a lack of long-term perspective and proactive management of prehospital resources. Many of the respondents describe an absence of governing documents on how different procedures should be carried out. Other things also highlighted here are financial arrangements for this work and lack of understanding from decision-makers, other parts of the healthcare system and other actors. Prehospital work differs in different places in the country and there are few prehospital medical services in Sweden, which creates competition for physicians interested in working in prehospital care.

“I think the biggest challenge is, what is the normal routine? What is a common way of working or approaching this challenge?” (1).

The category *knowledge and skills* highlight the importance of having a certain level of knowledge to be able to do the job. Here, the respondents also highlight how the work has changed with more experience and how things that were important when they were new to the profession become unimportant with more experience. Disorders that you have not seen so often involve challenges, even if they are not time-critical, due to the fact that you are unfamiliar and have limited experience in the field. Research in prehospital medicine is limited and there are few managers in prehospital medicine who have research competence.

“Yes, with that is that you feel uncomfortable, you really have to go back to your first years as a physician to remember how to look in the ears of children, that’s really it. It is a bit outside our comfort zone, even if it does not stress in that way. ” (10).

The organization prehospital is fundamentally different from that in hospital and just as the environment changes the conditions for the physicians, the organization sets limitations to what is possible prehospital. These limitations contribute to why decisions need to be made with limited information which in turn results in stress and a higher burden on the *emotional management*. One of the most obvious organizational challenges is the limited resources both in terms of equipment and the number of staff, which in turn puts a lot of pressure on the physicians to be able to manage.

## Discussion

The purpose of this study was to identify the challenges that physicians face when working in the prehospital environment. The main conclusions are presented here. Through our GT approach, we can illustrate how the consistent interplay of maintaining a balance between various factors impacts physicians in their prehospital work. We found no inherent hierarchy among the different themes. The analysis of the interviews showed that challenges are based on the complexity of the prehospital environment. The consequences of the extreme environment result in conflicting challenges, where the organizational pressures are set against the individual’s skills. This means that physicians struggle with, among other things, limited information support, limited resources, and the lack of colleagues to receive help from. The organizational conditions are fundamentally different in the prehospital environment compared to what is available in hospitals. The organizational conditions in prehospital care limit the physician’s opportunities both for decisions and for procedures, which places a great strain on the physicians’s individual abilities.

Another balancing act is the passive and the active leadership whereby the physicians constantly move between passive and the active leadership depending on the situation. Their individual abilities in the form of leadership and emotion management are both affected by and affect the environment they are in. Leadership is an interaction with the individuals in the environment where they are, both patients and colleagues.

### The prehospital environment

It is not only physicians who work in the prehospital environment; other professions also work in the prehospital environment in Sweden. Threats of violence from people under the influence of drugs and alcohol, affects the stress levels of the prehospital staff [2]. Among ambulance nurses, it has been reported that over 80% have been exposed to threats and violence when working prehospital [12]. In addition to threats and violence, there are also challenges in maintaining competence and confidence in how to handle situations that can be called rare events, for example diseases that are rarely seen. There is a time factor that is also challenging, where many patients need to get to the hospital quickly to get adequate care [13–15]. This creates a lot of pressure on physicians to prioritize between time and procedures. This type of prioritization creates an additional level of complexity compared to the decisions they make in hospitals. One of the ways in which the physicians describe they handle these challenges can be found in the category of reflexive leadership, where they describe the process of reflecting individually and as a team on the tasks they have undertaken. That physicians in Sweden do not exclusively work in prehospital settings also has a clear connection to the knowledge challenge. The idea is that by regularly working in hospitals, physicians maintain their clinical knowledge, even regarding less common conditions, due to the higher volume of patients they encounter in hospital settings.

Another component of the environment that is the same for ambulance staff and physicians alike is the culture involving loyalty but also an attitude that it does not matter what you have just been through, you should continue to work as if nothing happened. The work culture of hardiness has also been found among paramedics [16]. This part of the culture in the prehospital environment is of course closely linked to emotional management. What distinguishes the physicians’ work from the ambulance staff’s and what makes their situation unique is that physicians are expected to be able to manage and also solve situations that are problematic for ambulance staff, so the extreme situations for the ambulance staff become the everyday situation for the physician-staffed units.

### Organizational conditions

Although the basis of the work is the same in the hospital as prehospital, some differences create great challenges for the physicians and working alone is one of them. Of course, working alone does not mean that they work alone prehospital; they work in teams with others, above all nurses and in certain situations staff from the rescue service or the police. Carrying the formal liability for the situation is a source of stress that can be observed in many professions working in prehospital care [17]. At the hospital, doctors work together to a greater extent, especially when it comes to seriously ill or injured patients, and they can then share the burden of the responsibility. In addition to the responsibility itself, there are certain medical procedures that only physicians may perform, which means a great responsibility to be sufficiently competent to perform these procedures. The equipment used prehospital has clear limitations compared to what is available in hospitals. There is both a limited amount of equipment and the equipment is affected by the prehospital environment, which means that it stops working or works less well [18]. This shows the connection between the prehospital environment and in this case loneliness and emotional management. This explains why the level of both knowledge and skills are required to be on a higher level when working in the prehospital environment.

### Teamwork

Teamwork can be described as the integration of individuals’ efforts towards a common goal [19]. Teamwork is found everywhere in society, from sports teams to the military and of course also in healthcare. Although there are differences between these teams, for example how they are shaped or what abilities different individuals are expected to have, there are also several similarities [20]. Three abilities have been identified that can be seen regardless of which team you study. The first of these is coordination, which means that you use the team’s different resources in a wise way. This also includes that the team creates a common goal, which contributes to the team having a shared mental model. The second common ability is communication, which means that the various individuals in the team have the ability to both give and receive information. The last of the three common abilities is adaptability, encompassing the ability to adapt strategies and behaviors depending on changed conditions for the team. By participating in reflections and evaluations, the individuals in the team can develop their understanding of how they can adapt to different circumstances [20]. In addition to these three abilities, it has also been found that a “psychological safety” is required, for the individuals in the team to feel safe enough to express their opinions, which is necessary for the team to be able to develop when, for example, there is a conflict [20]. The skills needed for well-functioning teamwork have been shown to be the same in different environments and in different medical teams [21].

### Leadership

Leadership is identified as one of the most important abilities for the person responsible for the care of severely injured patients [22]. In this study, there were several challenges within the individual premise of leadership. One of the clearest challenges was how physicians needed to switch between different leadership roles. Switching between an active and a passive leadership role has also been seen during transport of patients within the hospital, where the physician alternates between leading the situation to being a consultant to anothers [23]. Being able to switch between roles is considered an ability to be able to adapt to different situations [24]. We found that the choice of leadership role depended a lot on how serious or time-critical the patient’s condition was and how the rest of the staff could cope with that situation, in other words the environment and the organizational conditions. The most effective leadership style varies depending on how severely injured the patient is and depending on how experienced the team that takes care of the patient is [25]. More direct leadership is more effective when the patient is severely injured and when the team is inexperienced. If alternatively the team is more experienced and the patient is not as severely injured, a supportive / inclusive leadership style is more effective [25].

There are many challenges in the communication between physicians and nurses that to some extent come from the different cultures within the two professional groups [26]. In this study, we found that the physicians are aware of their communication and use it in their leadership to create a positive atmosphere despite the fact that the situation is challenging. They invite other staff in decision-making by asking questions when the situation allows it. But just as they switch between different leadership roles, communication changes depending on the situation and there is an awareness that communication changes.

### Decision-making

The decision-making process that physicians face is limited by various factors that we have summarized in this theory: in the environment, organization, leadership, and emotion management. If you look at how Brehmer [27] describes what limits the scope for action in a rescue operation, you can see great similarities with our findings. Brehmer describes the limiting factors as; “Task, time, resources, accident, legal framework and environment” [27]. There were several things that were challenging in the decision-making for the physicians, the time factor being one of them, another being the changing environment. If you look at the four basic elements in what characterizes a dynamic decision-making, this correlates with our findings with the four basic elements being; that a series of decisions is required, that decisions are not independent and that the environment changes both spontaneously and as a consequence of the decision - maker’s actions [27].

It is clear that the decision-making process is challenging for the physicians and they describe how they use self-leadership to deal with these challenges. They control their thoughts to try to handle the challenge they face. This type of self-leadership can reduce anxiety in the situation they are in [28]. There was a reflexive leadership where the reflection on the leadership was both a process together with the team but also self-reflection where there were challenges in evaluating one’s own efforts and seeing alternative solutions. This type of self-awareness requires that you are prepared to immerse yourself in your own emotional world [29].

### Emotional management

The individual prerequisite for emotional management consisted of challenges in dealing with both one’s own emotions and of those around you. There was a very clear connection to the environment and its challenges as well as the organization with the limitations that exist in personnel and equipment. Workplace stress usually consists of job stress or interpersonal stress [30]. Situations can be divided into threat states or challenge states. In the first state it is perceived that the requirements exceed the resources and in the latter the situation is perceived as challenging but manageable. The challenge that comes from being responsible created stress where, among other things, situations arose where physicians were forced to decide which patient to prioritize creating a moral stress. Moral stress is when you are forced to make decisions that do not agree with your moral perception [31] or where there are two choices that are morally correct but by choosing one, the other becomes impossible [32]. The stress reactions in moral stress are essentially the same as in other stress reactions, but also consist of something termed “moral stress reactions” which include feelings of powerlessness, meaninglessness and inadequacy [32].

In the theme of emotional management, stress reactions also arose, both the reactions in connection with challenging events and reactions that persisted after the event. Immediate reactions in the form of heart rate increase and increased focus were not only described as something negative. The more experienced physicians described missing that feeling as it had become rarer with time. Larsson et al. [32] have shown that the long-term effects of stress on healthcare professionals are affected by the coping strategy of “emotional distancing”, which was something that also emerged in this study. In particular, it was described how it was more difficult to shield oneself emotionally in the prehospital environment because the physicians came so close to the patients when they took care of them in their homes. This could contribute to the physicians who work prehospital having a greater risk of negative effects in the long term due to stress. Posttraumatic stress has been found to be more prevalent in ambulance personnel compared to the rest of the population although there is a gender difference [17, 33]. Interestingly, the opposite has been found when looking at teams with physicians [34].

The physicians in our study took responsibility for the management of emotions not only for themselves but also for patients and relatives and for the rest of the team. It was often a matter of managing their own feelings to facilitate the situation for the others, for example to convey peace and security even if it was not their real feelings. Staying calm during an advanced procedure is important. If a physician is unable to manage their own feelings, it can affect how the rest of the team perceive them, This can impact their willingness to work with the physician in the future [35]. The physicians also took responsibility for the rest of the team after challenging events and took the time to talk and give praise for the others’ efforts. This is where emotional management and leadership overlap.

The word “challenge” was chosen with care; it contains of course a lot of difficulties but also something that attracts some people. It is not a completely negative word, and we can find it in the theme of emotional management. The study shows that the physicians who work prehospital are attracted there because of the excitement and challenge of working prehospital.

### Limitations

Grounded Theory methodology relies heavily on researcher interpretation, potentially introducing subjective biases into the analysis process. One limitation in our study is that one of the researchers possess significant prior knowledge, potentially influencing data interpretation. To counteract this, analysis was done collaboratively with the other researcher with no preconceptions.

Convenience sampling rely on readily accessible participants which may introduce selection bias, as individuals who volunteer or are easily available may not represent the broader population. Critical viewpoints or experiences that exist outside the easily accessible participant pool may be overlooked and potentially limiting the depth and richness of the data collected. Findings may therefor lack generalizability and may not accurately reflect the diversity of perspectives of prehospital physicians. We only had one female physician in the study, unfortunately this represents the lack of female physicians in prehospital care in Sweden.

The open-ended nature of semi structured interviews may result in incomplete or inconsistent responses, hindering the researcher’s ability to generate comprehensive and coherent theoretical frameworks. To mitigate this limitation, we opted for a single interviewer to conduct all interviews, aiming to maintain consistency in probing techniques and depth across interviews. The absence of a pilot interview to test the interview questions prior to data collection represents another limitation. Without a preliminary test interview, the efficacy and appropriateness of the interview questions remain unverified, potentially affecting the depth and quality of data obtained.

## Conclusion, suggestions for further research and practical implications

This is a study of physicians’ challenges when working prehospital. Although the physicians had the opportunity to define what they found challenging, all of them emphasized non-technical skills as the most challenging part of their work. This article contributes with valuable knowledge in a scientific field that is rarely studied and also in a professional group that is rarely studied.

In the prehospital environment, the individual conditions must be balanced against the organizational conditions. Physicians must, both as individuals and as part of a team, maneuver in time and space in terms of decision-making and practical work. Leadership alternates between passive and active leadership and they must balance their own and others’ emotional states.

Future studies may explore the themes of this study to create an understanding of leadership and emotional management in the prehospital environment. The themes and codes found in this study could also be used to look at challenges in a broader perspective in prehospital care and in an international prehospital environment.

With the help of previous studies and what we have found, it is reasonable to review what training is needed before starting to work prehospital as a physician. This should include components of the themes we have described: organization, environment, leadership and emotional management. In many professions where leadership and emotional management are important abilities and requirements, tests are also carried out to get a better idea of ​​the applicants’ abilities. Since we can show that there are a number of challenges in leadership and emotion management and that there is also much competition for the few prehospital positions, it is not unreasonable for employers to try to select the physicians they think have the best traits to be able to handle these challenges by using appropriate assessment tools.

### Electronic supplementary material

Below is the link to the electronic supplementary material.

Supplementary Material 1

## Data Availability

The data generated during and/or analyzed during the current study are not publicly available due to the data can only be distributed to the research group according to the ethical approval but limited information is available from the corresponding author on reasonable request.

## Abbreviations

GT: Grounded Theory
NTS: Non-technical skills
