# Supplementary material for: Physicians’ challenges when working in the prehospital environment - a qualitative study using grounded theory
Source: Int J Emerg Med. 2024 Feb 27;17:28. doi: 10.1186/s12245-024-00599-0 (PMC10900586; doi:10.1186/s12245-024-00599-0)
Supplement: Supplementary file 1 — Supplementary Material 1 [file 12245_2024_599_MOESM1_ESM.docx]

Interview guide

Background questions:

•Age

• Gender

• Years as a doctor

• Years as a pre-hospital doctor

**Theme 1 Organizational challenges:**

**•** What does a regular day look like for you?

• What is a challenge for you in your work?

• What challenges are there in the prehospital contextual/situational environment?

• Are the challenges in the prehospital environment different from how it usually is in the hospital?

• What are the expectations of doctors in these challenges?

• What role do the others play in these challenges?

• What is the difference when the rest of the team experiences the situation as challenging and when you experience the situation as challenging?

**Theme 2 Contextual/situational External challenges:**

**•** Are there any tricks you use to reduce the challenge of the situation?

• Is there anything you do to reduce the challenge for others involved in the situation?

• What opportunities do you have to influence the situation? Can you make it less challenging?

**Theme 3 personal Inner challenges:**

**•** How do you experience challenging situations?

• Do you have any tricks you use to manage yourself?

• Are there things you avoid when you find yourself in challenging situations?

• How do you manage your emotions after a challenging situation?

**Theme 4 medicine**

• Are there things in the prehospital environment that you find challenging?

**•** Which medical situations do you experience as challenging pre-hospital?

• What do you do to deal with these situations?

• Is there any equipment that causes difficulties in this environment?

• What do you do to deal with these difficulties with the equipment?
